# Supplementary material for: Identification of photoperiod-induced specific miRNAs in the adrenal glands of Sunite sheep (Ovis aries)
Source: Front Vet Sci. 2022 Jul 22;9:888207. doi: 10.3389/fvets.2022.888207 (PMC9354845; doi:10.3389/fvets.2022.888207)
Supplement: Supplementary file 1 [file Data_Sheet_1.ZIP › Supplementary file/miRDeep2 file.zip!/miRDeep2 file/Know_Structure/SP42A1/oar-let-7c.pdf]

miRBase precursor : oar-let-7c
Total read count : 76369
oar-let-7c read count : 76317
remaining reads : 52

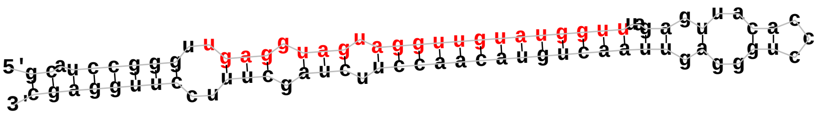

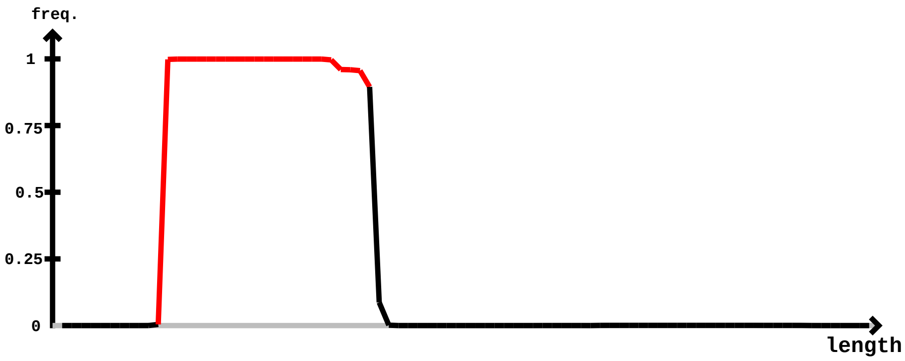


```
                oar-let-7c
5'- gcauccggguugagguaguagguuguaugguuuagaguuacacccugggaguuaacuguacaaccuucuagcuuuccuuggagc   -3'    exp
    ((.((((((..(((.(((.(((((((((((((..((.(..((...))..).))))))))))))))).))).)))..))))))))    reads      mm         sample
    ........guugagguaguagguuguaugg......................................................    2          0          seq
    ........guugagguaguagguuguauggu.....................................................    3          0          seq
    ........guugagguaguagguuguaugguu....................................................    3          0          seq
    .........uugagguaguagguuguaugg......................................................    4          0          seq
    .........uugagguaguagguuguauggu.....................................................    91         0          seq
    .........uugagguaguagguuguauAgu.....................................................    2          1          seq
    .........uugagguaguagguuguaugguu....................................................    212        0          seq
    .........uugagguaguagguuguauAguu....................................................    4          1          seq
    .........uugagguaguagguuguaugguuu...................................................    16         0          seq
    .........uugagguaguagguuguaugguuua..................................................    1          0          seq
    ..........uUagguaguagguugua.........................................................    1          1          seq
    ..........ugagguaguaAguugua.........................................................    2          1          seq
    ..........ugagguaguagguugua.........................................................    210        0          seq
    ..........ugagguUguagguugua.........................................................    1          1          seq
    ..........ugagguagGagguugua.........................................................    22         1          seq
    ..........ugagguaguaAguuguau........................................................    14         1          seq
    ..........ugagguUguagguuguau........................................................    7          1          seq
    ..........ugagguaguagguuguau........................................................    2455       0          seq
    ..........ugaAguaguagguuguau........................................................    13         1          seq
    ..........ugagguaguagguuUuau........................................................    16         1          seq
    ..........ugagguagGagguuguau........................................................    255        1          seq
    ..........ugagguaguagguuUuaug.......................................................    1          1          seq
    ..........ugagguaguagguuguaug.......................................................    66         0          seq
    ..........ugagguaguagguuguaugg......................................................    256        0          seq
    ..........ugagguaguagguuguauggu.....................................................    4546       0          seq
    ..........ugagguaguagguuguaugguu....................................................    61410      0          seq
    ..........ugagguaguagguuguaugguuu...................................................    6494       0          seq
    ..........ugagguaguagguuguauAguuua..................................................    64         1          seq
    ..........ugagguaguagguuguaugguuua..................................................    44         0          seq
    ..........ugagguaguagguuguauAguuuag.................................................    1          1          seq
    ...........gagguaguagguuguau........................................................    1          0          seq
    ...........gagguaguagguuguaugg......................................................    1          0          seq
    ...........gagguaguagguuguauggu.....................................................    7          0          seq
    ...........gagguaguagguuguaugguu....................................................    78         0          seq
    ...........gagguaguagguuguaugguuu...................................................    8          0          seq
```

oar-let-7c

gcauccgggu**ugagguaguagguuguaugguu**uagaguuacacccugggaguuaacuguacaaccuucuagcuuuccuuggagc			
...........gagguaguagguuguaugguuua..	1	0	seq
............agguaguagguuguaugguu..	2	0	seq
............agguaguagguuguaugguuu...	1	0	seq
.............gguaguagguuguaugguu..	2	0	seq
................................uagaguuacacccugggaguu...............................	1	0	seq
................................uagaguuacacccugggaguua..............................	1	0	seq
....gaguuaacuguacaaccuucuagc............	1	0	seq
....aguuaacuguacaaccuucuagcu...........	1	0	seq
....aacuguacaaccuucuagcuuu.........	1	0	seq
....cuguacaaccuucuagcuuu.........	3	0	seq
....cuguacaaccuucuagcuuuc........	6	0	seq
....cuguacaaccuucuagcuuucc.......	28	0	seq
....cuguacaaccuucuagcuuuccu......	8	0	seq
....cuguacaaccuucuagcuuuccuu.....	1	0	seq
....uguacaaccuucuagcuuuccu......	1	0	seq
